# Median duration and factors that influence the duration of symptom resolution in COVID‐19 patients in Ethiopia: A follow‐up study involving symptomatic cases

**DOI:** 10.1002/lim2.46

**Published:** 2021-08-21

**Authors:** Saro Abdella Abrahim, Masresha Tessema, Eshetu Ejeta, Mahammed Ahmed, Atkure Defar, Alemayehu Hussen, Getachew Demoz, Eskindir Degu, Mulugeta Aseratie, Belay Merga, Enatenesh Dillnessa, Tegene Regassa, Dereje Duguma, Susan Whiting

**Affiliations:** ^1^ Ethiopian Public Health Institute Addis Ababa Ethiopia; ^2^ Eka Kotebe General Hospital COVID‐19 Isolation and Treatment Center Addis Ababa Ethiopia; ^3^ The Ethiopian Ministry of Health Addis Ababa Ethiopia; ^4^ College of Pharmacy and Nutrition University of Saskatchewan Saskatoon Saskatchewan Canada

**Keywords:** COVID‐19, duration, symptom

## Abstract

**Background:**

Understanding the clinical features of COVID‐19 and duration for resolution of symptoms is crucial for isolation of patients and tailoring public health messaging, interventions and policy. Therefore, this study aims to assess the median duration of COVID‐19 signs and symptoms’ resolution and explore its predictors among symptomatic COVID‐19 patients in Ethiopia.

**Methods:**

A hospital‐based prospective cohort study involving 124 COVID‐19 cases was conducted at Eka Kotebe General Hospital, COVID‐19 Isolation and Treatment Center. The study participants were all symptomatic COVID‐19 adult patients admitted to the hospital from 18 March to 20 August 2020. Physicians at the centre recorded the data using a log sheet. Cox proportional‐hazards regression model was conducted. Statistical significance was defined at *P* < 0.05.

**Results:**

A total of 124 symptomatic COVID‐19 patients with a mean age of 42 years (±17) were involved in the study. The median duration of symptom resolution of COVID‐19 was seven days with a minimum of two and a maximum of sixty‐eight days. Sex and body mass index (BMI) were statistically significant predictors of the symptom resolution. The hazard of having delayed sign or symptom resolution in males was 55% higher than in females (*P* = 0.039; CI: 0.22–0.96) and the hazard of delayed sign or symptom resolution in those with BMI ≥ 25 kg/m^2^ was 35% higher than in those with BMI < 25 kg/m^2^ (*P* = 0.041; CI: 0.44–0.98]).

**Conclusions:**

The median duration of COVID‐19 symptom resolution was seven days. Being male and/or having a BMI ≥ 25 kg/m^2^ were predictors of a delayed sign or symptom resolution time. Therefore, it is important to consider proportion of males and those with BMI ≥ 25 kg/m^2^ when preparing isolation and treatment centres. Males and individuals with BMI ≥ 25 kg/m^2^ shall also be given priority when shielding from the COVID‐19.

## 1 BACKGROUND

The newly identified virus, Severe Acute Respiratory Syndrome Coronavirus 2 (SARS‐CoV‐2) has claimed more than a million lives worldwide since it was first recognized in Wuhan, China in December 2019.^1^ Countries all over the world have put in place preventive public health strategies to mitigate the devastating impact of the disease on their health system.^2^

Transmission of the virus occurs by close contact through respiratory droplets, by direct contact with infected persons or by contact with contaminated objects and surfaces.^3^ Isolation of COVID‐19 cases is a control measure in many countries limiting the spread of the virus.

According to the Centers for Disease Control and Prevention (CDC) recommendation, patients should be discharged from isolation centres based on two consecutive negative real‐time reverse transcriptase polymerase chain reaction (rRT‐PCR) test results of nasopharyngeal swabs obtained at least 24 h apart, and resolution of respiratory symptoms and fever without the use of fever‐reducing medicine.^4^

There are various studies that illustrated the duration of viral shedding among COVID‐19 patients. According to some of these studies, the median duration of viral shedding ranged from eight to thirty days, whereas the longest duration ranged from thirty‐seven to forty‐seven days.^10^, ^11^, ^12^, ^13^, ^14^, ^15^, ^16^, ^17^

The duration of viral shedding is a potential indicator of the infectivity and transmissibility of epidemic diseases. It is also one of the significant criteria for discharge and provides vital information for effective infection prevention and control.^9^, ^18^ According to studies from Beijing and Wuhan, the median duration of recovery from the virus after symptoms’ resolution is two and half a day and eight days, respectively.^19^, ^20^ This indicates that patients continued to be virus positive even after the resolution of symptoms.

Some of the studies conducted in China and western countries revealed that older age, delayed initiation of antiviral treatment, highest temperature at admission, male sex, coronary heart disease (CHD), comorbidity and decreased albumin levels were among important factors that affect the duration of shedding of the virus.^11^, ^14^, ^16^, ^21^ Corticosteroid treatment has also been indicated as a predictor of longer duration of clearing of the virus from the body^12^; however, another study claimed that low‐to‐moderate dosage of corticosteroid had little effect on the duration of viral excretion.^11^

On 27 May 2020, World Health Organization (WHO) put forward a new criteria for discharging patients from isolation. The resolution of symptoms is taken as a criteria to discharge patients without laboratory tests. WHO recommends discharging symptomatic patients ten days after symptom onset, plus at least three days without symptoms (without fever and respiratory symptoms), and discharge of asymptomatic patients ten days after first positive test result without requiring further testing.^5^

Understanding the clinical features of COVID‐19 and duration for resolution of symptoms is crucial for isolation of patients and tailoring public health messaging, interventions and policy. Based on the report of the WHO–China mission on Corona Virus Disease 2019, the top five typical signs and symptoms include fever, dry cough, fatigue, sputum production and shortness of breath.^3^

In a study done among COVID‐19 patients, the mean duration of COVID‐19 symptoms of mild and moderate patients who eventually recovered was eleven and half a day with a standard deviation of ±5.7 .^6^ WHO reported that the median duration for the COVID‐19 symptoms to resolve is two weeks, whereas it takes three to six weeks for patients with severe or critical disease.^3^ Moreover, a study conducted among outpatients with COVID‐19 in a Multistate Health Care Systems Network in the United States reported that mild cases took at least two weeks for everyone to return to their baseline health. The duration of the symptoms’ resolution was affected by factors such as obesity, reporting of three or more chronic illnesses and psychiatric illnesses.^7^ In terms of factors associated with severe progression of COVID‐19, older age, male sex and presence of underlying diseases were reported in several studies.^6^, ^8^, ^9^

Studies in different context had showed different average COVID‐19 symptom resolution periods and its predictors. Therefore, this study aims to explore the duration and its predictors of the symptom resolution among symptomatic COVID‐19 patients in Ethiopia. The findings can be used to tailor effective public health messages and adopt strategies for treatment and prevention measures.

## 2 METHODS

### 2.1 Study design and settings

A hospital‐based prospective cohort study involving 124 COVID‐19 cases was conducted at Eka Kotebe General Hospital, COVID‐19 Isolation and Treatment Center. The Center is the first hospital designated to manage positive COVID‐19 cases in Ethiopia. It has the capacity of admitting 600 cases. During the study period, all laboratory‐confirmed COVID‐19 patients (positive for SARS‐CoV‐2 on rRT‐PCR) regardless of sign and/or symptom development status were admitted to the hospital for follow‐up and treatment.

In this study, the dependent variable was duration of symptom resolution, whereas independent variables were age, sex, comorbidities and body mass index (BMI).

### 2.2 Study participants

The study participants were all symptomatic COVID‐19 adult patients admitted to the Eka Kotebe General Hospital, COVID‐19 Isolation and Treatment Center from 18 March to 20 August 2020. All COVID‐19 cases who manifested/reported any illness were invited to participate in the study. Recovered symptomatic participants’ data were considered for analysis instead of data of those deceased.

### 2.3 Sampling and study period

All symptomatic COVID‐19 cases who were admitted to the Eka Kotebe General Hospital, COVID‐19 Isolation and Treatment Center during the study period and who gave consent to participate in the study were included. The study enrolled cases and followed up from March to August 2020.

The symptomatic case was defined as any SARS‐CoV‐2‐positive person diagnosed by rRT‐PCR with at least one sign or symptom for COVID‐19. The signs and symptoms included, but not limited to, cough, fever, headache, muscle pain and shortness of breath.

### 2.4 Data collection

A log sheet to record patients’ symptom status was prepared. Physicians at the Eka Kotebe General Hospital, COVID‐19 Isolation and Treatment Center recorded the COVID‐19 signs and symptoms status of all patients every day. On‐site data entry was conducted using tablets by trained data collectors in the facility, and data were transferred to the Ethiopian Public Health Institute Server through the REDCap system.

Patients were asked to report their date of sign/symptom onset and resolution. The first date a patient experienced any kind of illness related to COVID‐19 was documented as the date of sign/symptom onset. The symptom resolution date was patient's first date free from any sign or symptom. The date of recovery was considered as the second SARS‐CoV‐2‐negative result date of their nasopharyngeal or throat swab.

### 2.5 Data management and analysis

Continuous variables were expressed as mean ± standard deviation (SD) for the normally distributed data or median with interquartile range (IQR) for the skewed data. Descriptive analysis of survival data was presented graphically using Kaplan–Meier estimator. Log‐rank test was used to compare the survival experience of different categories of covariates. The proportional hazard assumption was checked using Schoenfeld residual test. Cox proportional‐hazards regression model was used to determine the potential risk factors associated with the time to sign and symptom resolution among symptomatic COVID‐19 cases. Statistical significance was defined as *P* < 0.05. All analyses were done using STATA version 16.1 software.

### 2.6 Ethical clearance

The study protocol was developed by the study team and reviewed by the Ethiopian Public Health Institute's Institutional Review Board. The protocol was approved (Ethics Ref. No. EPHI 6.13/690). Informed consent from every participating case was obtained. Data security and participants’ confidentiality were maintained at all levels of data management. All methods were performed in accordance with the relevant guidelines and regulations.

### 2.7 Definitions

The following definitions are according to the National Institute for Health (NIH) treatment guideline updated on April 2021:

- Mild Illness: Individuals who have any of the various signs and symptoms of COVID‐19 (e.g. fever, cough, sore throat, malaise, headache, muscle pain, nausea, vomiting, diarrhoea, loss of taste and smell) but who do not have shortness of breath, dyspnoea or abnormal chest imaging.
- Moderate Illness: Individuals who show evidence of lower respiratory disease during clinical assessment or imaging and who have an oxygen saturation (Spo_2_) ≥94% on room air at sea level.
- Severe Illness: Individuals who have Spo_2_ < 94% on room air at sea level, a ratio of arterial partial pressure of oxygen to fraction of inspired oxygen (Pao_2_/Fio_2_) <300 mm Hg, respiratory frequency >30 breaths/min or lung infiltrates >50%.
- Critical Illness: Individuals who have respiratory failure, septic shock and/or multiple organ dysfunction.

## 3 RESULTS

A total of 124 symptomatic COVID‐19 patients with a mean age of 42 years (±17) were followed up to see symptom or sign resolution date. The majority (76%) of participants were male. The higher proportion of the participants (66%) had BMI < 25 kg/m^2^, and 29% of them had comorbidity (Table 1).

**Table 1:** Background information of the study participants (*N* = 124)

Variables	Category	Frequency (*n*)	Percent (%)
Age	Mean = 42 years ± 17 Min = 17, Max = 95		
Sex	Female	30	24
	Male	94	76
BMI	Mean = 24.2 kg/m^2^ ± 4.2 Min = 16.9, Max = 50.4		
BMI category	<25 kg/m^2^	82	66
	≥25 kg/m^2^	42	34
Comorbidity	Have no comorbidity	88	71
	Have at least one comorbidity	36	29

Thirty‐six patients had reported to have at least one pre‐existing medical condition. The most predominant pre‐existing medical conditions were non‐communicable diseases, particularly, Diabetes Mellitus and Hypertension. The median duration of symptom resolution in those with and without comorbidities was similar (Table 2).

**Table 2:** Reported comorbidities and median duration of symptom resolution (*N* = 36)

Comorbidity	Frequency	Median days of symptom resolution	IQR
HIV/AIDS	4	10	19.5
Diabetes	18	5.5	2
Heart disease	3	5	3
Hypertension	18	5	5
Asthma	3	6	5
Chronic liver disease	5	6	3
Chronic kidney disease	2	5.5	3
Cancer	6	7	3

**FIGURE 1:**
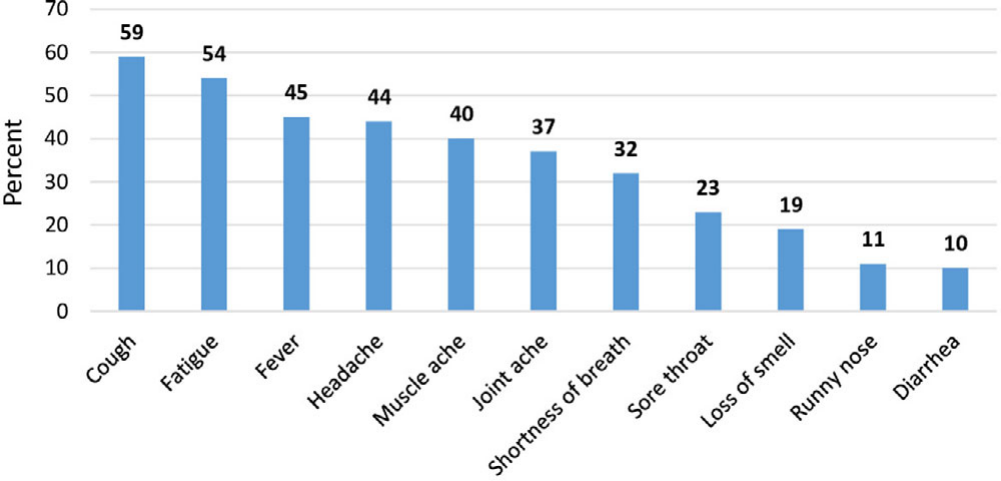
Percentage of signs and symptoms of COVID‐19 patients

Respiratory and neurological illnesses were among the most commonly reported signs and symptoms in the study participants. The majority of the participants (59%) had cough and only a few had reported gastrointestinal illness (10%) (Figure 1)

**FIGURE 2:**
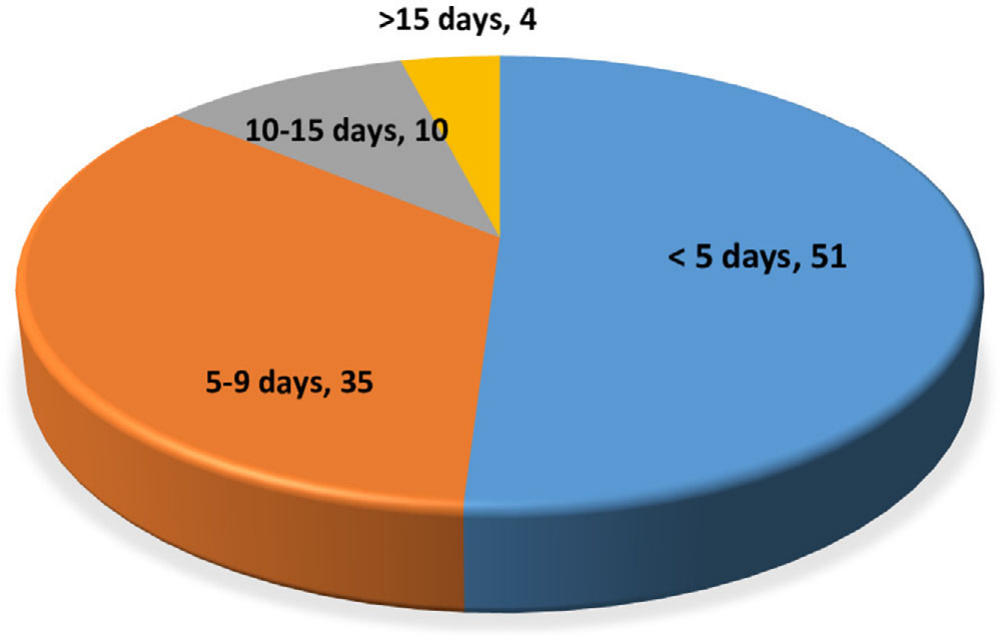
Proportion of participants and date of diagnosis

All the study participants had signs or symptoms before the date of diagnosis for SARS‐CoV‐2 by rRT‐PCR. The median duration between the date of diagnosis and date of sign or symptom onset was four days, ranging from one to twenty‐three days. The majority of the participants (51%) were diagnosed with the virus in five days after the onset of signs or symptoms and 35% in five to nine days. Only 4% were diagnosed after fifteen days of sign or symptom onset (Figure 2).

**FIGURE 3:**
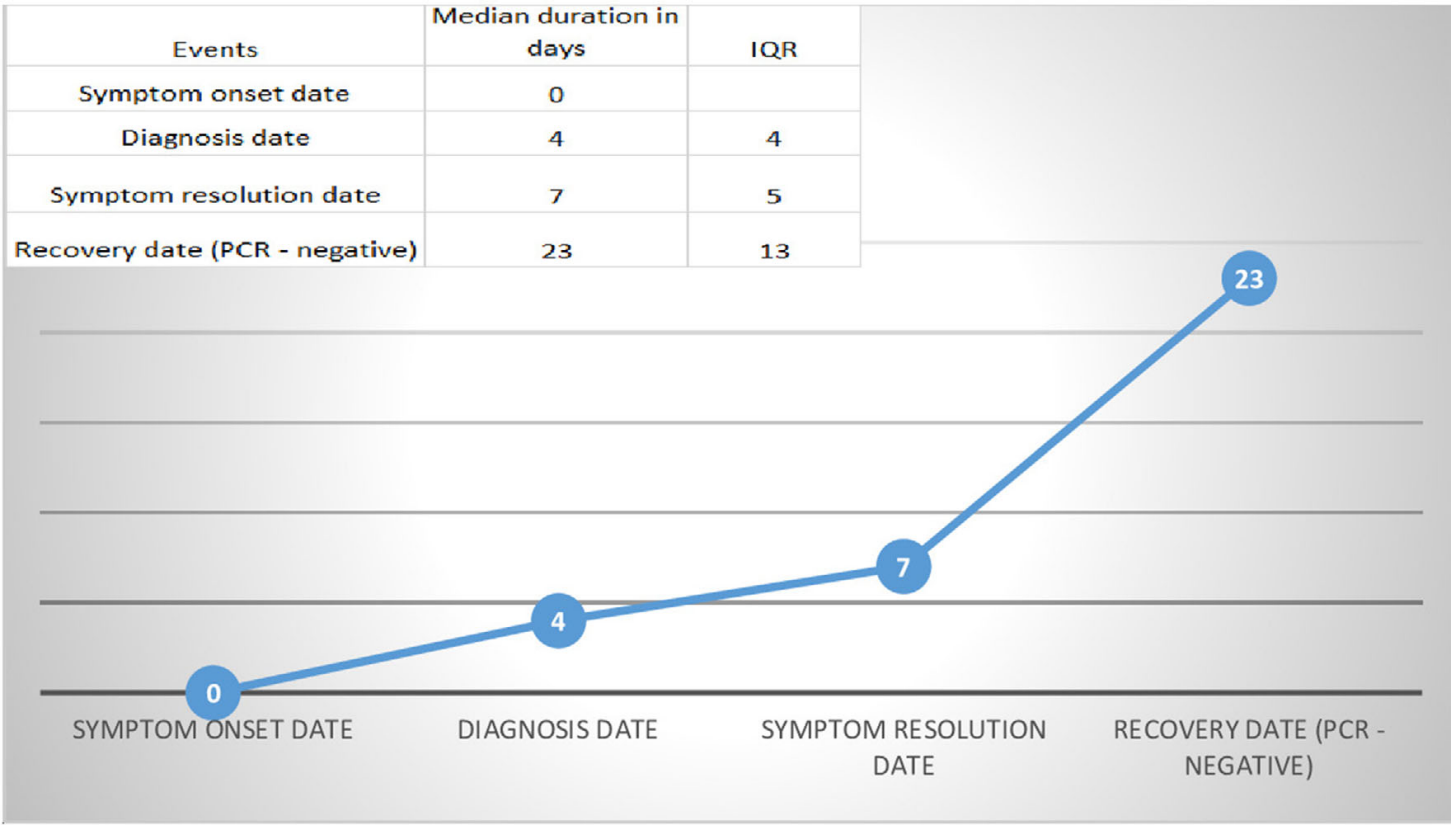
Median durations of symptoms and recovery

The median duration of identification of COVID‐19 patients in community screening was four days after being symptomatic. The median duration of the symptoms’ resolution was seven days (IQR = 5). The median duration of recovery from the virus, rRT‐PCR‐negative test for SARS‐CoV‐2, was sixteen days (IQR = 13) after symptom resolution (Figure 3).

The median duration of sign or symptom resolution of COVID‐19 in the study participants was seven days with a minimum of two and a maximum of sixty‐eigh days. The median duration of symptom resolution in males was eight days, whereas it was six days in females. Participants with BMI ≥ 25 kg/m^2^ took a longer duration to get free of their respective signs or symptoms compared with those with BMI < 25 kg/m^2^. Duration of symptom resolution in those with and without comorbidity was similar.

Controlling for age and comorbidity, sex was found to be a significant predictor of sign or symptom resolution. The hazard of having delayed sign or symptom resolution in males was 55% higher than in females (*P* = 0.039; CI: 0.22–0.96) and the hazard of the delayed sign or symptom in those with BMI ≥ 25 kg/m^2^ was 35% higher than in those with BMI < 25 kg/m^2^ (*P* = 0.041; CI: 0.44–0.98). Age and comorbidity had no association with a delayed sign or symptom in COVID‐19 patients (Table 3).

**Table 3:** Predictors of duration of symptom resolution among COVID‐19 patients: Cox proportional‐hazards analysis (*n* = 124)

Variables	Categories	Duration of symptom resolution^b^	Log‐rank test, *P*‐value	AHR (95% CI)	*P*‐value AHR
Age	42 years ± 17			0.99 (0.97, 1.01)	0.611
Sex	Male	8	0.269	0.45 (0.22, 0.96)	0.039^b^
	Female	6		1	
Comorbidity	No comorbidity	7		1	
	At least one comorbidity	7	0.926	0.69 (0.33, 1.44)	0.32
BMI	<25 kg/m^2^	6		1	
	≥25 kg/m^2^	8	0.051	0.65 [0.44, 0.98]	0.041^b^

^a^ Symptom resolution duration was defined as duration from the first date of first sign or symptom onset to the last date of any sign or symptom.

^b^ When adjusted for age and comorbidity, sex and BMI were significant determinant of signs or symptoms’ resolution.

## 4 DISCUSSION

In this study, we have determined the median duration of symptoms’ resolution of COVID‐19 and explored predictors of symptom resolution among symptomatic COVID‐19 patients in Ethiopia. The median duration of sign or symptom resolution of COVID‐19 in the study participants was 7 days with a minimum of 2 and a maximum of 68 days. Controlling for influencing covariates, sex and BMI were found to be significant predictors of sign or symptom resolution. In this study, age and comorbidity had no association with a delayed sign or symptom in COVID‐19 patients.

According to Chang et al.^19^ and Siordia Jr. et al.,^22^ the commonly reported symptoms in patients with COVID‐19 were fever, cough, and dyspnoea. Headache, loss of smell and nasal obstruction were the most common clinical presentations of mild to moderate patients in Europe,^6^ whereas fever, cough, dyspnoea and fatigue were the most common clinical presentations in Asian.^23^, ^24^ Our study also reported a similar finding in which cough, fever, headache and sore throat were among the most common signs and symptoms. Furthermore, the finding of this study showed that Diabetes Mellitus and hypertension were the most common comorbidities among study participants. Hypertension, diabetes mellitus and cardiovascular disease were the most commonly reported comorbidities according to a study done on the clinical features of COVID‐19.^22^

The median duration of symptom resolution in our study is comparable to a study done in Beijing, China by Chang et al. that reported a median duration of 8 days (IQR = 6.25–11.5).^19^ However, this study's median duration is lower than the median duration of a study done in Soonchunhyang University Seoul Hospital (ten days with a minimum of two and a maximum of thirty‐eight days),^25^ in European hospitals (eleven and half a day with a standard deviation of 5.7)^6^ and in the hospitals outside Wuhan (eleven days).^26^ Difference in genetics, sociodemographic status, patient's disease status and sample size might be the possible reasons for the differences observed in the median duration of symptom resolution in the above‐mentioned studies and ours.

There is yet little information on the predictors of duration of COVID‐19 signs and symptoms’ resolution; however, the finding of this study revealed that sex and BMI were associated with the duration of COVID‐19 symptoms’ resolution. The hazard of having delayed sign or symptom resolution in males was more likely than in females. The reason might be that females are less likely to have complications related to viral infections because of innate immunity, steroid hormones and sex hormones.^6^ However, a study conducted on symptom duration and a risk factor for delayed return to usual health among outpatients with COVID‐19 in the United States reported that there was no significant association between sex and return to usual health.^7^

COVID‐19 patients with BMI ≥ 25 kg/m^2^ have delayed duration of COVID‐19‐related sign or symptom resolution. This finding is similar to the study done in the United States and reported that those with a higher BMI were twice less likely to return to usual health state as compared with those with lower BMI (<30 kg/m^2^).^7^ A retrospective study conducted on COVID‐19 patients in New York City had also revealed that obese (BMI ≥ 30 kg/m^2^) patients had a significantly higher rate of intensive care unit admission or death.^27^ Moreover, a dose–response meta‐analysis study conducted on BMI and outcome in patients with COVID‐19 indicated that BMI ≥ 30 kg/m^2^ was associated with mortality and severity in patients with COVID‐19.^28^

This study showed that age was not significantly associated with the duration of COVID‐19 sign or symptom resolution. However, a study conducted in the United States showed that older age was associated with delayed return to their usual health state.^7^ The age‐dependent defects in T‐cell and B‐cell function and excess production of type 2 cytokines lead to a deficiency in control of viral replication and more prolonged pro‐inflammatory responses, which in turn lead to poor outcome.^9^, ^29^ A prospective cohort study conducted on critically ill patients with laboratory‐confirmed COVID‐19 patients in New York City also reported that older age patients were more likely to develop progressive COVID‐19 and die.^30^ In our study, the average age of participants was 42 years with a standard deviation of ±17 and the age of 95% of the participants was less than 65 years. This could mask the possible association between age and symptom resolution. Our study is the first published work in Africa to report the average duration and predictors of the symptom resolution of COVID‐19.

### 4.1 Limitations of the study

Although using prospective follow‐up study is the strength of this study, the results shall be interpreted with caution. There could be a chance that duration of mild symptoms might be overlooked by individuals and not reported. In addition, the average age of participants in this study was 42 years. Apart from small sample size, the relatively younger age group in this study compared to the other populations in the Western world should be taken into consideration when comparing and interpreting the findings.

## 5 CONCLUSION

Our findings showed that the median duration of COVID‐19 sign or symptom resolution was seven days. Being male and/or having a BMI ≥ 25 kg/m^2^ were predictors of delayed sign or symptom resolution period. An average of two weeks were required to be cured of SARS‐CoV‐2 for symptomatic patients after symptom resolution. Therefore, it is important to consider proportion of males and/or those with BMI ≥ 25 kg/m^2^ when preparing isolation and treatment centres. Males and patients with BMI ≥ 25 kg/m^2^ shall also be given priority when shielding from the COVID‐19.

## ETHICS APPROVAL AND CONSENT TO PARTICIPATE

The study protocol was developed by the study team and reviewed by the Ethiopian Public Health Institute's Institutional Review Board. The protocol was approved (Ethics Ref. No. EPHI 6.13/690).

All participants had provided informed consent to participate in the study. The lead authors affirm that the manuscript is an honest, accurate and transparent account of the study being reported; that no important aspects of the study have been omitted; and that any discrepancies from the study as planned have been explained.

## AVAILABILITY OF DATA AND MATERIALS

The data set used and analysed during this study will be made available from the corresponding author on reasonable request.

## CONFLICT OF INTEREST

The authors declare no conflict of interest.

## AUTHOR CONTRIBUTIONS

SA and MT designed the study. SA, MT, EE, AD and AH performed the statistical analysis and wrote the first draft of the manuscript. MAA, GD, ESD, MUA, BM and END contributed further to the development of the manuscript to the scientific standards. TR, DD and SW supervised the whole process of the study and proof read the manuscript. All authors read and approved for correspondence to publication.

## References

[1] WHO . Clinical Management of COVID‐19: Interim Guidance . World Health Organization; 2020.

[2] Güner R , Hasanoğlu I . COVID‐19: prevention and control measures in community. Turk J Med Sci. 2020;50(3):571‐577. 32293835 10.3906/sag-2004-146 PMC7195988

[3] WHO . Report of the WHO‐China Joint Mission on Coronavirus Disease 2019 (COVID‐19) . World Health Organization; 2020.

[4] CDC . Discontinuation of Transmission‐Based Precautions and Disposition of Patients with COVID‐19 in Healthcare Settings (Interim Guidance) . Centers for Disease Control and Prevention; 2021.

[5] WHO . Criteria for Releasing COVID‐19 Patients from Isolation: Scientific Brief . World Health Organization; 2020.

[6] Lechien JR , Chiesa‐Estomba CM , Place S , et al. Clinical and epidemiological characteristics of 1420 European patients with mild‐to‐moderate coronavirus disease 2019. J Intern Med. 2020;288(3):335‐344. 32352202 10.1111/joim.13089 PMC7267446

[7] Tenforde MW , Kim SS , Lindsell CJ , et al. Symptom duration and risk factors for delayed return to usual health among outpatients with COVID‐19 in a multistate health care systems network ‐ United States, March‐June 2020. MMWR Morb Mortal Wkly Rep. 2020;69(30):993‐998. 32730238 10.15585/mmwr.mm6930e1 PMC7392393

[8] Voinsky I , Baristaite G , Gurwitz D . Effects of age and sex on recovery from COVID‐19: analysis of 5,769 Israeli patients. J Infect. 2020;81(2):E102‐E103. 10.1016/j.jinf.2020.05.026 PMC7229949 32425274

[9] Zhou F , Yu T , Du R , et al. Clinical course and risk factors for mortality of adult inpatients with COVID‐19 in Wuhan, China: a retrospective cohort study. Lancet. 2020;395(10229):1054‐1062. 32171076 10.1016/S0140-6736(20)30566-3 PMC7270627

[10] Chen X , Zhu B , Hong W , et al. Associations of clinical characteristics and treatment regimens with viral RNA shedding duration in patients with COVID‐19. Int J Infect Dis. 2020;98:252‐260. 32619760 10.1016/j.ijid.2020.06.091 PMC7326382

[11] Shi D , Wu W , Wang Q , et al. Clinical characteristics and factors associated with long‐term viral excretion in patients with severe acute respiratory syndrome coronavirus 2 infection: a single‐center 28‐day study. J Infect Dis. 2020;222(6):910‐918. 32614392 10.1093/infdis/jiaa388 PMC7337834

[12] Li TZ , Cao ZH , Chen Y , et al. Duration of SARS‐CoV‐2 RNA shedding and factors associated with prolonged viral shedding in patients with COVID‐19. J Med Virol. 2021;93(1):506‐512. 32644223 10.1002/jmv.26280 PMC7362127

[13] Warabi Y , Tobisawa S , Kawazoe T , et al. Effects of oral care on prolonged viral shedding in coronavirus disease 2019 (COVID‐19). Spec Care Dentist. 2020;40(5):470‐474. 32706510 10.1111/scd.12498 PMC7405138

[14] Yan D , Liu XY , Zhu YN , et al. Factors associated with prolonged viral shedding and impact of lopinavir/ritonavir treatment in hospitalised non‐critically ill patients with SARS‐CoV‐2 infection. Eur Respir J. 2020;56(1):2000799. 32430428 10.1183/13993003.00799-2020 PMC7241115

[15] Qi L , Yang Y , Jiang D , et al. Factors associated with duration of viral shedding in adults with COVID‐19 outside of Wuhan, China: a retrospective cohort study. Int J Infect Dis. 2020;96:531‐537. 32425636 10.1016/j.ijid.2020.05.045 PMC7231495

[16] Zheng X , Chen J , Deng L , et al. Risk factors for the COVID‐19 severity and its correlation with viral shedding: a retrospective cohort study. J Med Virol. 2021;93(2):952‐961. 32725915 10.1002/jmv.26367 PMC7821149

[17] van Kampen JJ , van de Vijver DA , Fraaij PL , et al. Shedding of infectious virus in hospitalized patients with coronavirus disease‐2019 (COVID‐19): duration and key determinants. Nat Commun. 2021;12(1):267. 33431879 10.1038/s41467-020-20568-4 PMC7801729

[18] Ryoo SM , Kim WY , Sohn CH , et al. Factors promoting the prolonged shedding of the pandemic (H1N1) 2009 influenza virus in patients treated with oseltamivir for 5 days. Influenza Other Respir Viruses. 2013;7(5):833‐837. 23279949 10.1111/irv.12065 PMC5781218

[19] Chang D , Mo G , Yuan X , et al. Time kinetics of viral clearance and resolution of symptoms in novel coronavirus infection. Am J Respir Crit Care Med. 2020;201(9):1150‐1152. 32200654 10.1164/rccm.202003-0524LE PMC7193851

[20] Fu Y , Han P , Zhu R , et al. Risk factors for viral RNA shedding in COVID‐19 patients. Eur Respir J. 2020;56(1):2001190. 32398298 10.1183/13993003.01190-2020 PMC7236829

[21] Hu F , Yin G , Chen Y , et al. Corticosteroid, oseltamivir and delayed admission are independent risk factors for prolonged viral shedding in patients with Coronavirus Disease 2019. Clin Respir J. 2020;14(11):1067‐1075. 32750201 10.1111/crj.13243 PMC7436608

[22] Siordia JA Jr . Epidemiology and clinical features of COVID‐19: a review of current literature. J Clin Virol. 2020;127:104357. 32305884 10.1016/j.jcv.2020.104357 PMC7195311

[23] Cao Y , Liu X , Xiong L , Cai K . Imaging and clinical features of patients with 2019 novel coronavirus SARS‐CoV‐2: a systematic review and meta‐analysis. J Med Virol. 2020;92(9):1449‐1459. 32242947 10.1002/jmv.25822 PMC7228215

[24] Huang C , Wang Y , Li X , et al. Clinical features of patients infected with 2019 novel coronavirus in Wuhan, China. Lancet. 2020;395(10223):497‐506. 31986264 10.1016/S0140-6736(20)30183-5 PMC7159299

[25] Park SY , Yun SG , Shin JW , et al. Persistent sever acute respiratory syndrome coronavirus 2 detection after resolution of coronavirus disease 2019‐associated symptoms/signs. Korean J Intern Med. 2020;35(4):793‐796. 32549526 10.3904/kjim.2020.203 PMC7373965

[26] Xu K , Chen Y , Yuan J , et al. Factors associated with prolonged viral RNA shedding in patients with COVID‐19. Clin Infect Dis. 2020;71(15):799‐806. 32271376 10.1093/cid/ciaa351 PMC7184421

[27] Hajifathalian K , Kumar S , Newberry C , et al. Obesity is associated with worse outcomes in COVID‐19: analysis of Early Data From New York City. Obesity. 2020;28(9):1606‐1612. 32470210 10.1002/oby.22923 PMC7283831

[28] Pranata R , Lim MA , Yonas E , et al. Body mass index and outcome in patients with COVID‐19: a dose‐response meta‐analysis. Diabetes Metab. 2021;47(2):101178. 32738402 10.1016/j.diabet.2020.07.005 PMC7388778

[29] Opal SM , Girard TD , Ely EW . The immunopathogenesis of sepsis in elderly patients. Clin Infect Dis. 2005;41(Suppl. 7):S504‐S512. 16237654 10.1086/432007

[30] Cummings MJ , Baldwin MR , Abrams D , et al. Epidemiology, clinical course, and outcomes of critically ill adults with COVID‐19 in New York City: a prospective cohort study. Lancet. 2020;395(10239):1763‐1770. 32442528 10.1016/S0140-6736(20)31189-2 PMC7237188

